# Anti-leukemic activity of bortezomib and carfilzomib on B-cell precursor ALL cell lines

**DOI:** 10.1371/journal.pone.0188680

**Published:** 2017-12-13

**Authors:** Kazuya Takahashi, Takeshi Inukai, Toshihiko Imamura, Mio Yano, Chihiro Tomoyasu, David M. Lucas, Atsushi Nemoto, Hiroki Sato, Meixian Huang, Masako Abe, Keiko Kagami, Tamao Shinohara, Atsushi Watanabe, Shinpei Somazu, Hiroko Oshiro, Koshi Akahane, Kumiko Goi, Jiro Kikuchi, Yusuke Furukawa, Hiroaki Goto, Masayoshi Minegishi, Shotaro Iwamoto, Kanji Sugita

**Affiliations:** 1 Department of Pediatrics, School of Medicine, University of Yamanashi, Chuo, Japan; 2 Department of Pediatrics, Graduate School of Medical Science, Kyoto Prefectural University of Medicine, Kyoto, Japan; 3 College of Pharmacy, The Ohio State University, Columbus, OH, United States of America; 4 Stem Cell Regulation, Center for Molecular Medicine, Jichi Medical School, Shimotsuke, Japan; 5 Hematology/Oncology & Regenerative Medicine, Kanagawa Children’s Medical Center, Yokohama, Japan; 6 Tohoku Block Center, Japanese Red Cross Society, Sendai, Japan; 7 Department of Pediatrics, Mie University Graduate School of Medicine, Tsu, Japan; University of South Alabama Mitchell Cancer Institute, UNITED STATES

## Abstract

Prognosis of childhood acute lymphoblastic leukemia (ALL) has been dramatically improved. However, prognosis of the cases refractory to primary therapy is still poor. Recent phase 2 study on the efficacy of combination chemotherapy with bortezomib (BTZ), a proteasome inhibitor, for refractory childhood ALL demonstrated favorable clinical outcomes. However, septic death was observed in over 10% of patients, indicating the necessity of biomarkers that could predict BTZ sensitivity. We investigated *in vitro* BTZ sensitivity in a large panel of ALL cell lines that acted as a model system for refractory ALL, and found that Philadelphia chromosome-positive (Ph+) ALL, *IKZF1* deletion, and biallelic loss of *CDKN2A* were associated with favorable response. Even in Ph-negative ALL cell lines, *IKZF1* deletion and bilallelic loss of *CDKN2A* were independently associated with higher BTZ sensitivity. BTZ showed only marginal cross-resistance to four representative chemotherapeutic agents (vincristine, dexamethasone, l-asparaginase, and daunorubicin) in B-cell precursor-ALL cell lines. To improve the efficacy and safety of proteasome inhibitor combination chemotherapy, we also analyzed the anti-leukemic activity of carfilzomib (CFZ), a second-generation proteasome inhibitor, as a substitute for BTZ. CFZ showed significantly higher activity than BTZ in the majority of ALL cell lines except for the P-glycoprotein-positive t(17;19) ALL cell lines, and *IKZF1* deletion was also associated with a favorable response to CFZ treatment. P-glycoprotein inhibitors effectively restored the sensitivity to CFZ, but not BTZ, in P-glycoprotein-positive t(17;19) ALL cell lines. P-glycoprotein overexpressing ALL cell line showed a CFZ-specific resistance, while knockout of P-glycoprotein by genome editing with a CRISPR/Cas9 system sensitized P-glycoprotein-positive t(17;19) ALL cell line to CFZ. These observations suggested that *IKZF1* deletion could be a useful biomarker to predict good sensitivity to CFZ and BTZ, and that CFZ combination chemotherapy may be a new therapeutic option with higher anti-leukemic activity for refractory ALL that contain P-glycoprotein-negative leukemia cells.

## Introduction

Bortezomib (BTZ) is a proteasome inhibitor approved for the treatment of multiple myeloma (MM) [1]. Recently, BTZ has been suggested as a new therapeutic option for acute lymphoblastic leukemia (ALL) treatment [2]. *In vitro* anti-leukemic activity of BTZ against ALL was firstly reported in 2000 [3]. Subsequently, a clinical case report revealed that administration of BTZ followed by dexamethasone (Dex) induced transient clinical response in a childhood ALL patient suffering from multiple relapses [4], and, in another study, BTZ monotherapy demonstrated favorable outcome in a xenograft ALL model [5]. However, a phase 1 study showed that BTZ was ineffective against recurrent or refractory pediatric ALL as a single agent [6]. In contrast, BTZ had synergistic or additive cytotoxic effects on ALL cell lines *in vitro* when combined with standard chemotherapeutic agents [7]. Based on these findings, combination therapy with BTZ and the standard chemotherapy platform of vincristine (VCR), dexamethasone (Dex), pegylated asparaginase (Asp), and doxorubicin was conducted by the TACL (Therapeutic Advances in Childhood Leukemia & Lymphoma) consortium. A phase 1 study in children with relapsed ALL demonstrated promising results [8], and a following phase 2 study revealed the effectiveness of BTZ combination chemotherapy in refractory childhood ALL [9]: 16 (73%) of 22 patients achieved complete remission (CR) or CR without platelet recovery. 20 of 22 patients were B-cell precursor ALL (BCP-ALL) patients, and their response rate was 80% (16 of 20 patients). Although BTZ combination chemotherapy was effective, severe side effects were notable: 10 patients (45.5%) experienced severe infection and three septic deaths (13.6%) were reported. Thus, it is important to identify biomarkers that can predict response to BTZ in clinical practice. Moreover, to develop more effective and safer combination therapy, it is also important to clarify possible cross-resistance between BTZ and other chemotherapeutic agents.

Carfilzomib (CFZ), a second-generation proteasome inhibitor, demonstrated more potent and more specific proteasome inhibition against the chymotrypsin-like activity of the 20S proteasome in a stable and irreversible fashion [10–12]. CFZ also showed durable and less toxic activity as a single agent in patients with advanced MM [13–16]. In a recently reported randomized phase 3 study in relapsed MM patients, the outcome of combination therapy with Dex and CFZ was significantly better than that with BTZ [17, 18], suggesting that CFZ combination chemotherapy may be a more effective and safer therapeutic option for refractory ALL.

In the present study, we investigated the association of cytogenetic abnormalities with *in vitro* BTZ sensitivity and possible cross-resistance of BTZ with conventional chemotherapeutic agents using a large panel of ALL cell lines. We also tested the *in vitro* anti-leukemic activity of CFZ in ALL cell lines as a possible substitute for BTZ in BTZ combination chemotherapy for refractory ALL.

## Materials and methods

### Cell lines

Seventy-nine BCP-ALL cell lines, nine T-ALL cell lines, and two MM cell lines listed in S1 Table were analyzed. BCP-ALL cell lines included 14 Philadelphia chromosome-positive (Ph+) ALL cell lines, 11 MLL-rearranged (MLL+) ALL cell lines, 16 t(1;19)-ALL cell lines, 4 t(17;19)-ALL cell lines, 3 t(12;21)-ALL cell lines, and 31 B-others ALL cell lines. The group classified as “B-other” included BCP-ALL cell lines carrying none of the above representative five translocations. KOPN, KOCL, YAMN, and YACL series of cell lines were sequentially established in our laboratory from 1980 to 2011 as previously reported [19, 20]. YCUB and KCB series of cell lines were sequentially established at Yokohama City University and Kanagawa Children’s Medical Center [21] and were provided in 2014 (H. Goto). THP series of cell lines, L-KUM and L-ASK, were sequentially established at Tohoku University [22] and were provided in 2014 (M. Minegishi). MB series of cell lines were sequentially established at Mie University Graduate School of Medicine [23] and were provided in 2014 (S. Iwamoto). SU-Ph2 [24] was established at Kinki University School of Medicine, Osaka, and was provided in 2010 (Dr. Y. Maeda). TCCY [25] was established at Tochigi Cancer Center and was provided in 2011 (Dr. Y. Sato). HALO1 [20] and SK9 [26] were established at Tokyo Medical University, Tokyo, and were provided in 1997 (Dr. T. Look in Dana-Farber Cancer Institute, Boston, MA) and in 2012 (Dr. S. Okabe), respectively. Endokun [20] was established at Iwate Medical University, Morioka, and was provided in 1997 (Dr. M. Endo). Kasumi2 [27] was established at Hiroshima University, Hiroshima, and was provided in 2010 (Dr. T. Inaba). SCMCL1 and SCMCL2 [28] were established at Saitama Children’s Medical Center and were provided in 2014 (Dr. J. Takita). P30/OHK [29] and Nalm27 [30] were purchased from ATCC in 2012. To verify the significance of P-glycoprotein overexpression in resistance to CFZ, subline of 697 (697R) [31] that was established after long-term culture of 697 cells in the presence of stepwise increasing concentrations of silvestrol and its parental cells were analyzed. All cell lines were maintained in RPMI1640 medium supplemented with 10% fetal calf serum in a humidified atmosphere of 5% CO_2_ at 37°C.

### alamarBlue cell viability assay

To determine the 50% inhibitory concentrations (IC50s) of BTZ, CFZ, Dex, daunorubicin (DNR), VCR, and L-Asp, an alamarBlue cell viability assay (Bio-Rad Laboratories, Hercules, CA) was performed. 1–4 x 10^5^ cells were plated into a 96-well flat-bottom plate and assays performed in triplicate in the presence or absence of seven concentrations of each drug. The cells were cultured for 44 h for sensitivities to BTZ, CFZ, DNR, and VCR, or 68 h for sensitivities to Dex and L-Asp. After a 6 h additional incubation with alamarBlue, absorbance at 570 nm were monitored by a microplate spectrophotometer using 600 nm as a reference wavelength. Cell survival was calculated by expressing the ratio of the optical density of treated wells to that of untreated wells as a percentage. The concentration of agent required to reduce the viability of treated cells to 50% of untreated cells was calculated, and the median of three independent assays was determined as IC50 for each cell line.

### Flow cytometric analysis

To detect apoptosis, cells were cultured in the absence or presence of BTZ or CFZ for 17–18 h, stained with a fluorescein isothiocyanate (FITC)-conjugated Annexin-V and 7AAD (MBL, Nagoya, Japan). To verify combination of P-glycoprotein inhibitors, cells were pretreated with verapamil or nilotinib for 2 h, and subsequently cultured in the absence or presence of BTZ or CFZ for 17–18 h. Cell surface expression of P-glycoprotein was analyzed with a FITC-conjugated anti-P-glycoprotein antibody (Nichirei, Tokyo, Japan). For the functional assay of P-glycoprotein-mediated efflux of calcein-AM (CAM, abcam, Cambridge, UK), cells were incubated with 0.25 mM of CAM for 10 min at 37°C in the presence or absence of verapamil or nilotinib. To detect apoptosis in P-glycoprotein knockout HALO1 cells, cells were stained with a FITC-conjugated anti-P-glycoprotein antibody and a phycoerythrin (PE)-conjugated Annexin-V (MBL, Nagoya, Japan). The stained cells were analyzed by flow cytometry (FACSCalibur, BD Biosciences, San Jose, CA).

### Western blot analysis

Cells were solubilized in lysis buffer (50 mM Tris-HCl, pH 7.5, 150 mM NaCl, 1% Nonidet P-40, 5 mM EDTA, 0.05% NaN_3_, 1 mM phenylmethylsulfonyl fluoride, 100 μM sodium vanadate). The lysates were separated on a SDS-polyacrylamide gel under reducing conditions and then transferred to a nitrocellulose membrane. The membrane was incubated with the primary antibody at 4°C overnight, and then with horseradish peroxidase-labeled secondary antibody at room temperature for 1 hour. The bands were developed using an enhanced chemiluminescence detection (ECL) kit (Amersham Japan, Tokyo, Japan). Anti-CHOP (#2895, 1,000 x dilution) and anti-NOXA (ab13654, 500 x dilution) mouse monoclonal antibodies were purchased from Cell Signaling Technology (Danvers, MA) and abcam (Cambridge, UK), respectively. Horseradish peroxidase-conjugated anti-mouse IgG (pAb-HPR 330, 1,000 x dilution) was purchased from MBL (Nagoya, Japan).

### Gene copy number alteration

SALSA multiplex ligation-dependent probe amplification (MLPA) kit P335-A4 was used according to the manufacturer’s instructions (MRC Holland, Amsterdam, the Netherlands) [32]. The kit includes probes for *IKZF1*, *CDKN2A*, *CDKN2B*, *PAX5*, *ETV6*, *RB1*, *BTG1*, *EBF1*, and the *PAR1* region, which includes *CRLF2*, *CSF2RA*, and *IL3RA*. PCR fragments generated with the MLPA kit were separated by capillary electrophoresis on an ABI Prism 3130 Genetic Analyzer (Applied Biosystems, Foster City, CA). The relative copy number, obtained after normalization against controls, was used to determine genomic copy number of each gene. Values between 0.75 and 1.3 were considered to be within normal range. Values below 0.75 or 0.25 indicated monoallelic or biallelic loss, respectively.

### Knockout of P-glycoprotein with CRISPR-Cas9 system

To knock out P-glycoprotein (P-gp; ABCB1) expression with CRISPR/Cas9 system, we screened exon 3 of the *ABCB1* gene using the CRISPR design tool (CRISPR DESIGN, http://crispr.mit.edu). We selected 5’-tttggctgccatcatccatgg-3’, which showed the highest off-target hit score, and the synthesized oligomers were cloned into CRISPR/Cas9 vector (CRISPR CD4 Nuclease Vector, Thermo Fisher Scientific, Waltham, MA). Three days after electroporation of the *ABCB1*-targeting CRSIPR/Cas9 vector using Neon electroporation transfection system (Thermo Fisher Scientific) into HALO1 cells, CD4-positive cells were selected using CD4-microbeads (Miltenyi Biotec, Auburn, CA, USA) and expanded for further analyses.

### Statistics

We applied Mann-Whitney test for comparison of drug sensitivities and chi-square test for comparison of incidence in gene deletion. We applied a paired t-test for comparison of two means that were from the same objects and Pearson’s correlation analysis for correlation between paired data. All analyses were performed using Excel software. A multivariate analysis of log IC50 value of BTZ was performed to evaluate the effects of *IKZF1* deletion and biallelic loss of *CDKN2A* in 65 Ph-negative BCP-ALL cell lines using Excel software.

## Results

### Anti-leukemic activity of BTZ

**Fig 1:**
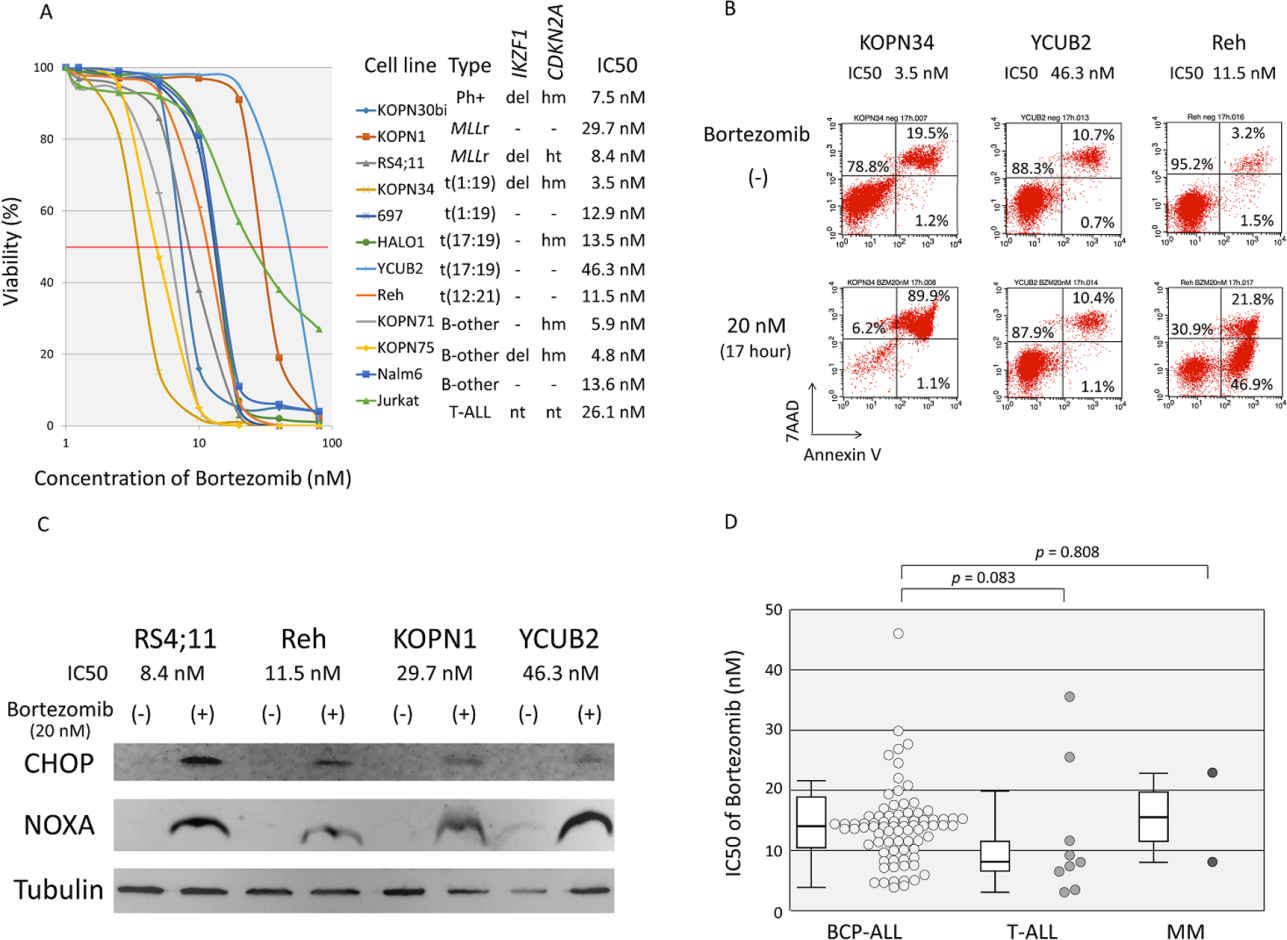
Anti-leukemic activity of bortezomib. (A) Dose-response curve of bortezomib sensitivity in representative cell lines. The vertical axis indicates % viability in alamarBlue cell viability assay and the horizontal axis indicates log concentration of bortezomib (nM). Phenotype, deletion of *IKZF1* and *CDKN2A*, and IC50 of each cell is indicated. Abbreviations: del, deletion; -, no deletion; hm, homozygous deletion; ht, heterozygous deletion; nt, not tested. (B) Induction of apoptotic cell death by bortezomib. Three cell lines (KOPN34, YCUB2 and Reh) were cultured in the presence or absence of 20 nM of bortezomib for 17 hours, and analyzed with Annexin V-binding (horizontal axis) and 7AAD-staining (vertical axis) using flow cytometry. The percentages of living cells (Annexin V-negative/7AAD-negative) and early (Annexin V-positive/ 7AAD-negative) and late (Annexin V-positive/ 7AAD-positive) apoptotic cells are indicated. (C) Induction of CHOP and NOXA expression. Highly sensitive cell lines (RS4;11 and Reh) and moderately sensitive cell lines (KOPN1 and YCUB2) were cultured in the presence or absence of bortezomib at 20 nM for eight hours, and immunoblotting was performed using Tubulin as an internal control. (D) Bortezomib sensitivity in 79 BCP-ALL, 9 T-ALL, and two MM cell lines. The vertical axis indicates the IC50 value for bortezomib. The *p* values determined by a Mann-Whitney test and boxplots are indicated.

We examined the *in vitro* sensitivity to BTZ using 79 BCP-ALL, 9 T-ALL, and 2 MM cell lines. Based on dose-response curves determined by the alamarBlue cell viability assay using seven concentrations of BTZ that ranged from 1.25 nM to 80 nM (Fig 1A), the IC50 for each cell line was determined. Induction of apoptosis was confirmed in representative cell lines by flow cytometric analyses (Fig 1B). BTZ has been shown to induce expression of CHOP, a critical transcriptional factor that can mediate apoptosis as a result of endoplasmic reticulum stress [33], and NOXA, one of the pro-apoptotic BH3-only members of the BCL2 family [34, 35]. We confirmed induction of CHOP and NOXA expression by a treatment with BTZ in both highly sensitive cell lines and in moderately sensitive cell lines (Fig 1C). The IC50 of BTZ was ranged from 3.1 nM to 46.3 nM, and the median IC50 was 13.5 nM (Mean ± SD: 13.9 ± 7.2 nM) (S2 Table). The median IC50s of BCP-ALL, T-ALL, and MM cell lines were 13.7 nM (13.9 ± 6.5 nM), 8.6 nM (12.9 ± 11.9 nM), 16.1 nM (16.1 ± 10.7 nM), respectively (Fig 1D). T-ALL cell lines tended to be more sensitive to BTZ than BCP-ALL cell lines (p = 0.083 by Mann-Whitney test), but there was no significant difference in BTZ sensitivity between BCP-ALL and MM cell lines (p = 0.808).

### Higher BTZ sensitivity in Ph+ALL cell lines

**Fig 2:**
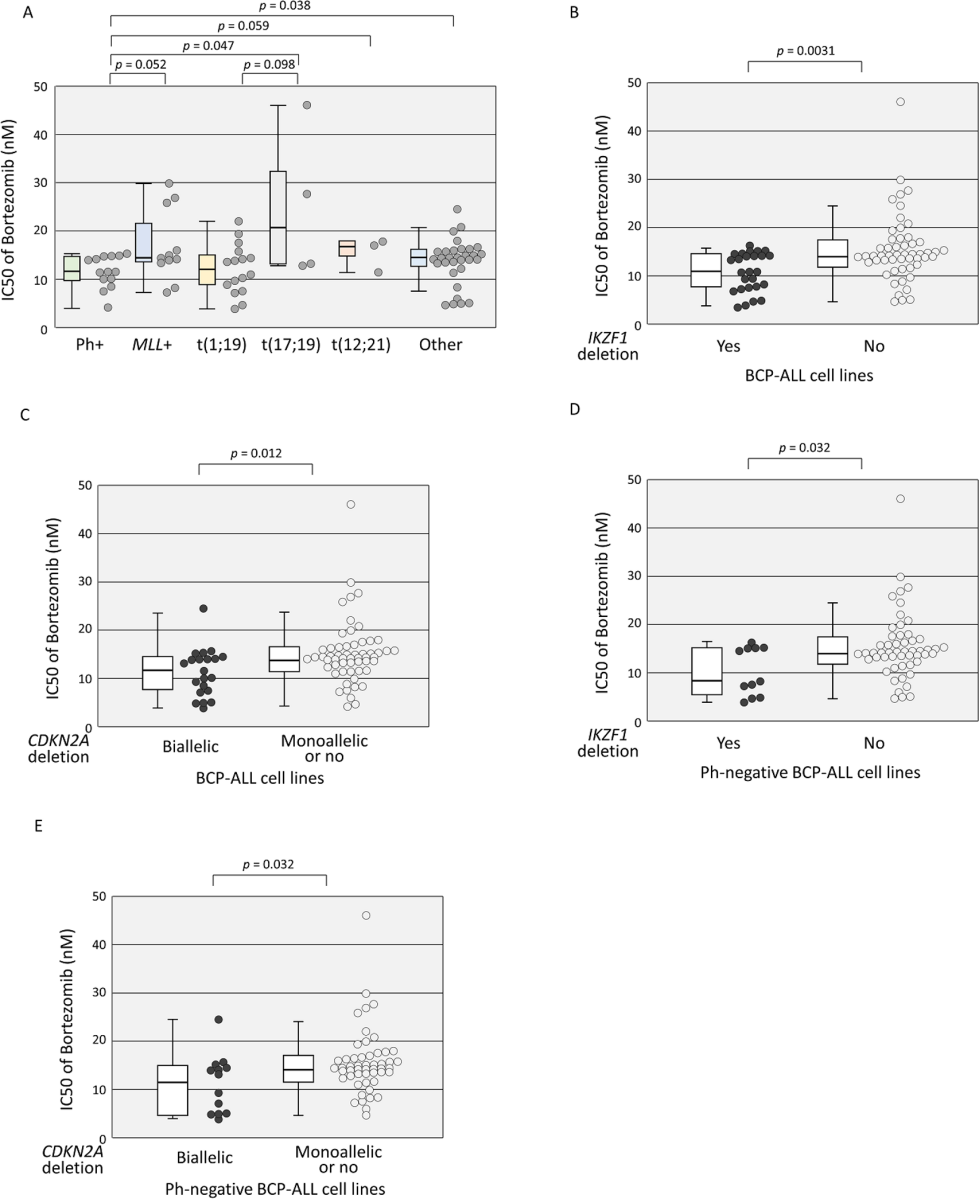
Anti-leukemic activity of bortezomib in BCP-ALL cell lines. (A) Association of bortezomib sensitivity with different types of translocation in BCP-ALL cell lines. The vertical axis indicates the IC50 value for bortezomib. Boxplot of each type is indicated. The *p* value determined by a Mann-Whitney test is indicated when < 0.1. (B-E) Associations of *IKZF1* deletion (B and D) and homozygous *CDKN2A* deletion (C and E) with bortezomib sensitivity in BCP-ALL cell liens (B and C) or in Ph-negative BCP-ALL cell lines (D and E). The vertical axis indicates the IC50 value for bortezomib. The *p* value determined by a Mann-Whitney test and boxplots are indicated.

We compared the sensitivity of 79 BCP-ALL cell lines with different types of translocations to BTZ, which included 14 Ph+ALL, 11 *MLL*+ALL, 16 t(1;19)-ALL, 4 t(17;19)-ALL, and 3 t(12;21)-ALL cell lines (Fig 2A and S2 Table). The remaining 31 BCP-ALL cell lines that did not carry any of the above five representative translocations were classified as “B-other”. Of note, Ph+ALL cell lines (median IC50: 11.4 nM; Mean ± SD: 11.4 ± 3.1 nM) were significantly more sensitive to BTZ than t(17;19)-ALL cell lines (20.8 nM; 25.2 ± 15.7 nM; p = 0.047 by Mann-Whitney test) and B-other ALL cell lines (13.9 nM; 13.4 ± 4.7 nM; p = 0.038), and tended to be more sensitive than *MLL*+ALL cell lines (14.6 nM; 16.7 ± 7.6 nM; p = 0.052) and t(12;21)-ALL cell lines (17.1 nM; 15.5 ± 3.5 nM; p = 0.059). No statistically significant difference was observed between Ph+ALL cell lines and t(1;19)-ALL cell lines (12.1 nM; 12.0 ± 5.2 nM; p = 0.547). Further, t(1;19)-ALL cell lines tended to be more sensitive to BTZ than t(17;19)-ALL cell lines (p = 0.098).

### Association of deletions of *IKZF1*, *CDKN2A*, and *CDKN2B* with Ph+ALL cell lines

We next investigated the association of BTZ sensitivity with copy number alteration of nine representative genes or region [32], since it has been reported that Ph+ALL frequently harbors deletions of *IKZF1* [36] and *CDKN2A*/*CDKN2B* [37] genes. Indeed, all 14 of the Ph+ALL cell lines carried deletions of *IKZF1*, *CDKN2A*, and *CDKN2B*, while 11 (16.9%), 36 (55.4%), and 40 (61.5%) out of 65 Ph-negative BCP-ALL cell lines carried deletions of *IKZF1*, *CDKN2A*, and *CDKN2B*, respectively (S3 Table). Deletion of *IKZF1* (p<0.00001 by chi-square test), *CDKN2A* (p = 0.0014), or *CDKN2B* (p = 0.0035) was significantly more common in Ph+ALL cell lines than in Ph-negative BCP-ALL cell lines. Regarding deletion of *CDKN2A*/*CDKN2B* genes, biallelic loss of *CDKN2A* was significantly more common in Ph+ALL cell lines (64.3%) than in Ph-negative BCP−ALL cell lines (20%; p = 0.0019), while there was no statistically significant difference in the incidence of biallelic loss of *CDKN2B* between Ph+ALL cell lines (42.9%) and Ph-negative BCP-ALL cell lines (26.6%; p = 0.196) (S3 Table).

### Association of *IKZF1* deletion and biallelic loss of *CDKN2A* with higher BTZ sensitivity

Among the nine genes or region tested, only deletion of *IKZF1* was significantly associated with higher BTZ sensitivity (Table 1). The IC50 of 25 BCP-ALL cell lines carrying *IKZF1* deletion (median IC50: 11.4 nM; Mean ± SD: 10.8 ± 4.0 nM) was significantly lower than that of 54 BCP-ALL cell lines not carrying the deletion (14.1 nM; 15.3 ± 7.0 nM; p = 0.0031 by Mann-Whitney test) (Fig 2B). Although deletions of *CDKN2A*/*CDKN2B* were significantly more common in Ph+ALL cell lines, the IC50s of 50 and 29 BCP-ALL cell lines with or without *CDKN2A* deletion (13.6 nM vs. 13.9 nM; 13.3 ± 5.5 nM vs. 15.0 ± 8.0 nM), respectively, were almost similar, and those of 54 and 25 BCP-ALL cell lines with or without *CDKN2B* deletion (13.7 nM vs. 13.7 nM; 13.6 ± 5.7 nM vs. 14.6 ± 8.2 nM), respectively, were identical (Table 1). When we focused on homozygous deletions, the IC50 of 22 BCP-ALL cell lines with biallelic loss of *CDKN2A* (12.2 nM; 11.2 ± 5.0 nM) was significantly lower than that of 57 BCP-ALL cell lines with monoallelic or no loss of *CDKN2A* (13.9 nM; 15.0 ± 6.8 nM; p = 0.012) (Fig 2C), while that of 22 BCP-ALL cell lines with biallelic loss of *CDKN2B* (13.6 nM; 13.1 ± 6.7 nM) was almost similar to that of 57 BCP-ALL cell lines with monoallelic or no loss of *CDKN2B* (13.9 nM; 14.2 ± 6.5 nM) (data not shown).

**Table 1:** Association of gene copy number alteration with BTZ-sensitivity in BCP-ALL cell lines.

Gene	Deletion		No deletion		*p* value
	N	Median IC50 (nM)	N	Median IC50 (nM)	Mann-Whitney test
*CDKN2B*	54	13.7	25	13.7	0.414
*CDKN2A*	50	13.6	29	13.9	0.483
*IKZF1*	25	11.4	54	14.1	0.0031
*PAX5*	21	13.1	58	13.8	0.571
PAR1*	17	13.9	62	13.7	0.463
*BTG1*	15	13.7	64	13.7	0.891
*ETV6*	10	11.8	69	13.9	0.247
*RB1*	5	13.7	74	13.7	0.732
*EBF1*	3	14.9	76	13.7	0.969

*PAR1 region includes CRLF2, CSF2RA, and IL3RA.

### *IKZF1* deletion and biallelic loss of *CDKN2A* as independent favorable factors for BTZ sensitivity

The above observations suggested that higher BTZ sensitivity of Ph+ALL cell lines may be associated with *IKZF1* deletion and/or biallelic loss of *CDKN2A*. Among 65 Ph-negative BCP-ALL cell lines, 11 cell lines carrying *IKZF1* deletion (median IC50: 8.4 nM; Mean ± SD: 10.0 ± 4.9 nM) were significantly more sensitive to BTZ than 54 cell lines without the deletion (14.1 nM; 15.3 ± 7.0 nM; p = 0.032 by Mann-Whitney test) (Fig 2D), and 13 cell lines carrying biallelic loss of *CDKN2A* (12.9 nM; 10.9 ± 6.2 nM) were significantly more sensitive than 52 cell lines with monoallelic or no loss of *CDKN2A* (14.3 nM; 15.3 ± 6.9 nM) (p = 0.032) (Fig 2E). Among 11 cell lines carrying deletion of *IKZF1*, only three cell lines harbored biallelic loss of *CDKN2A* (S1 Fig), suggesting that deletion of *IKZF1* and biallelic loss of *CDKN2A* were independent favorable factors for BTZ sensitivity in Ph-negative BCP-ALL cell lines. To further verify the significance of *IKZF1* deletion and biallelic loss of *CDKN2A*, we performed multiple regression analysis using the log IC50 values of BTZ in 65 Ph-negative BCP-ALL cell lines (Table 2). Of note, we found that *IKZF1* deletion (p = 0.004) and biallelic loss of *CDKN2A* (p = 0.007) were independently associated with higher BTZ sensitivity.

**Table 2:** Result of multiple regression analysis for association of IKZF1 deletion and bilalleleic loss of CDKN2A with BTZ-sensitivity in 65 Ph-negative BCP-ALL cell lines.

Variable	Hazard ratio	95% CI	*p* value
*IKZF1* deletion	-0.185	-0.312 to -0.060	0.004
Biallelic loss of *CDKN2A*	-0.165	-0.284 to -0.047	0.007

### Minimal cross-resistance of BTZ to conventional chemotherapeutic agents

**Fig 3:**
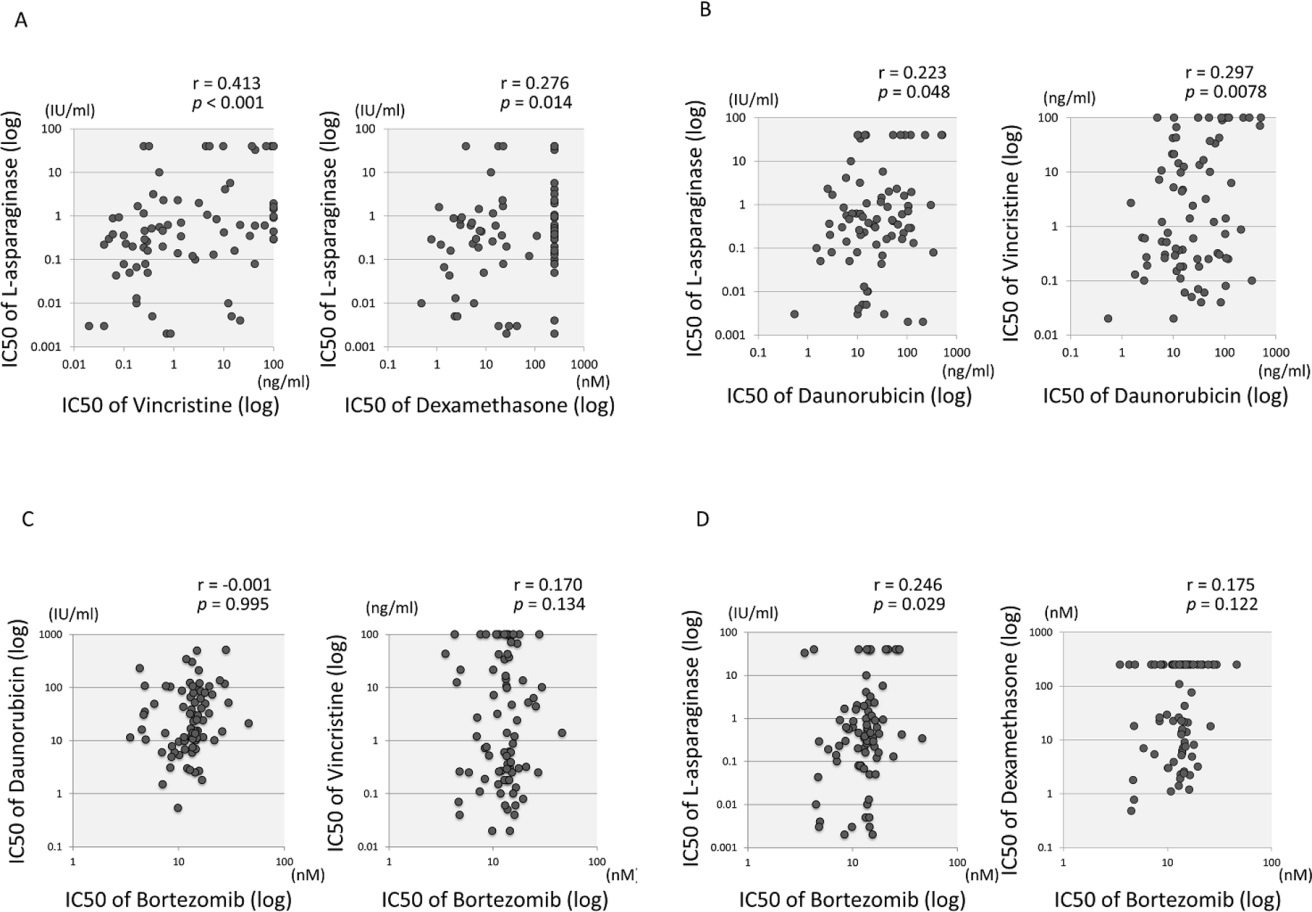
Cross-resistance of bortezomib and representative chemotherapeutic agents in 79 BCP-ALL cell lines. (A and B) Cross-resistance between two of four chemotherapeutic agents. Each vertical or horizontal axis indicates the log IC50 value for either of daunorubicin, vincristine, L- asparaginase, or dexamethasone. Correlation coefficients and *p* values are shown at the top of each panel. (C and D) Cross-resistance between bortezomib and the four chemotherapeutic agents. The vertical axes indicate the log IC50 values for daunorubicin (C), vincristine (C), L-asparaginase (D), and dexamethasone (D), whereas the horizontal axis indicates those for bortezomib. Correlation coefficients and *p* values are shown at the top of each panel.

To investigate cross-resistance of BTZ to the chemotherapeutic agents that were used in the BTZ combination therapy in the TACL study [8, 9], we examined the correlation between BTZ sensitivity and sensitivities to VCR, DNR, Dex, and L-Asp in 79 BCP-ALL cell lines (S4 Table, and Fig 3A and 3B). Sensitivity to L-Asp showed moderate (r = 0.413, p<0.001), weak (r = 0.276, p = 0.014), and marginal (r = 0.223, p = 0.048) correlation with that to VCR, Dex, and DNR, respectively, and sensitivity to DNR showed weak (r = 0.297, p = 0.0078) correlation with that to VCR. In contrast, sensitivity to BTZ did not show any significant correlation with the four drugs except for a very weak correlation with that to L-Asp (r = 0.246, p = 0.029) (S4 Table, and Fig 3C and 3D).

### *In vitro* anti-leukemic activity of CFZ

**Fig 4:**
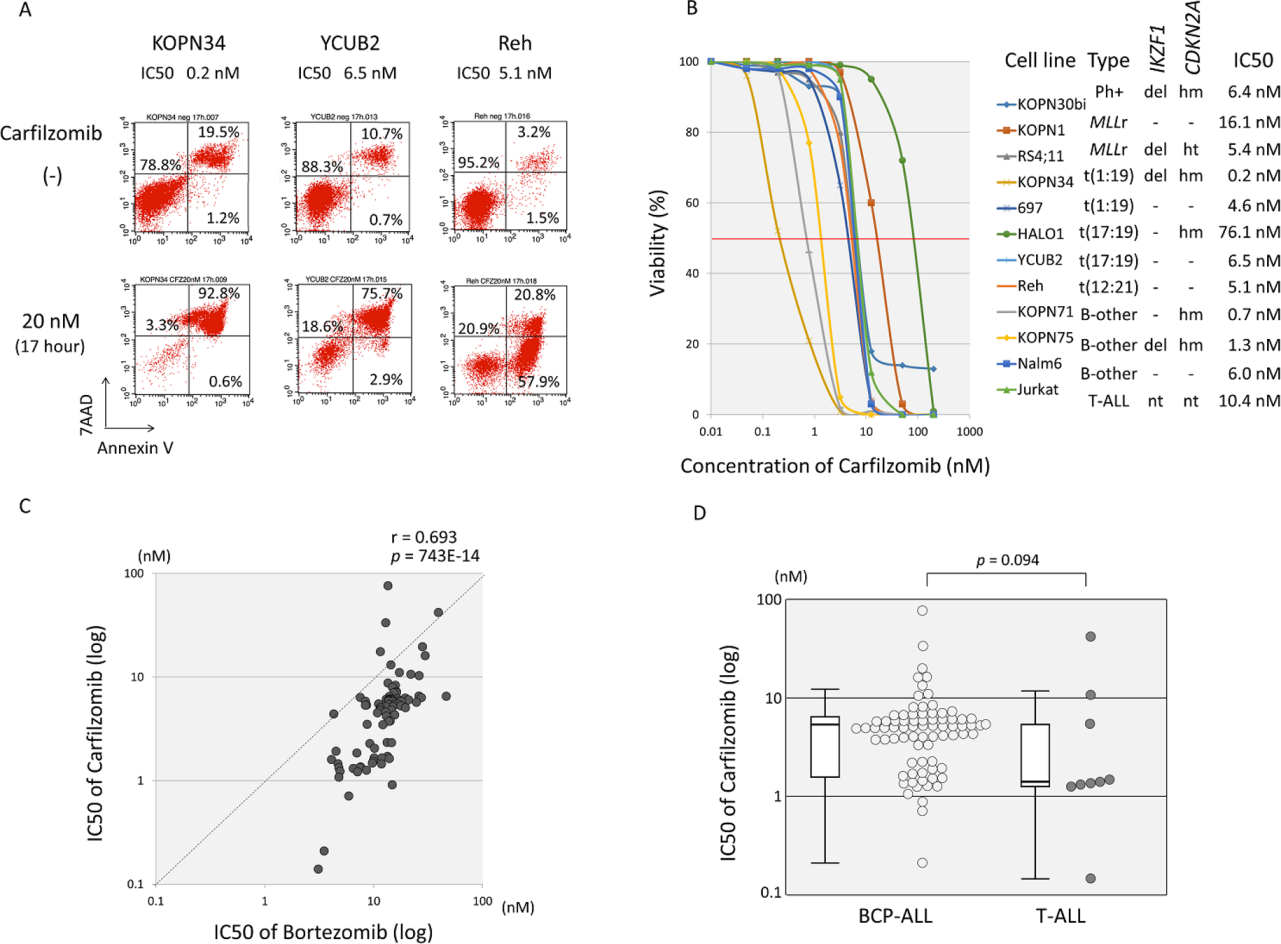
Anti-leukemic activity of carfilzomib. (A) Induction of apoptotic cell death by carfilzomib. Three cell lines (KOPN34, YCUB2 and Reh) were cultured in the presence or absence of 20 nM of carfilzomib for 17 hours, and analyzed with Annexin V-binding (horizontal axis) and 7AAD-staining (vertical axis) using flow cytometry. The percentages of living cells (Annexin V-negative/7AAD-negative) and early (Annexin V-positive/ 7AAD-negative) and late (Annexin V-positive/ 7AAD-positive) apoptotic cells are indicated. (B) Dose-response curve of carfilzomib sensitivity in representative cell lines. The vertical axis indicates % viability and the horizontal axis indicates the log concentration of carfilzomib (nM). Phenotype, deletion of *IKZF1* and *CDKN2A*, and IC50 of each cell is indicated. Abbreviations: del, deletion; -, no deletion; hm, homozygous deletion; ht, heterozygous deletion; nt, not tested. (C) Correlation of anti-leukemic activity of carfilzomib with that of bortezomib in 79 BCP-ALL cell lines and 9 T-ALL cell lines. The vertical axes indicate the log IC50 values for carfilzomib, whereas the horizontal axis indicates that for bortezomib. Correlation coefficient and *p* value are shown at the top of panel. (D) Carfilzomib sensitivity in 79 BCP-ALL cell lines and 9 T-ALL cell lines. The vertical axis indicates the IC50 value for carfilzomib. The *p* value determined by a Mann-Whitney test and boxplots are indicated.

We next investigated the *in vitro* anti-leukemic activity of CFZ, a second-generation proteasome inhibitor with irreversible activity [10]. Induction of apoptosis was confirmed in representative cell lines by flow cytometric analyses (Fig 4A). Based on the dose-response curves determined by the alamarBlue cell viability assay using seven concentrations of CFZ that ranged from 0.5 nM to 200 nM (Fig 4B), the IC50 was determined in 79 BCP-ALL and 9 T-ALL cell lines (S2 Table). The IC50 of CFZ ranged from 0.14 nM to 76.1 nM, and sensitivities to CFZ and BTZ were strongly correlated with each other; the correlation coefficient of the log IC50 values was 0.693 (p<0.0001) (Fig 4C). The median IC50s of BCP-ALL and T-ALL cell lines were 5.36 nM (Mean ± SD: 6.5 ± 9.2 nM) and 1.48 nM (7.2 ± 13.5 nM), respectively, and T-ALL cell lines tended to be more sensitive than BCP-ALL cell lines (p = 0.094 by Mann-Whitney test) (Fig 4D).

### Association of cytogenetic abnormalities with CFZ sensitivity in BCP-ALL cell lines

**Fig 5:**
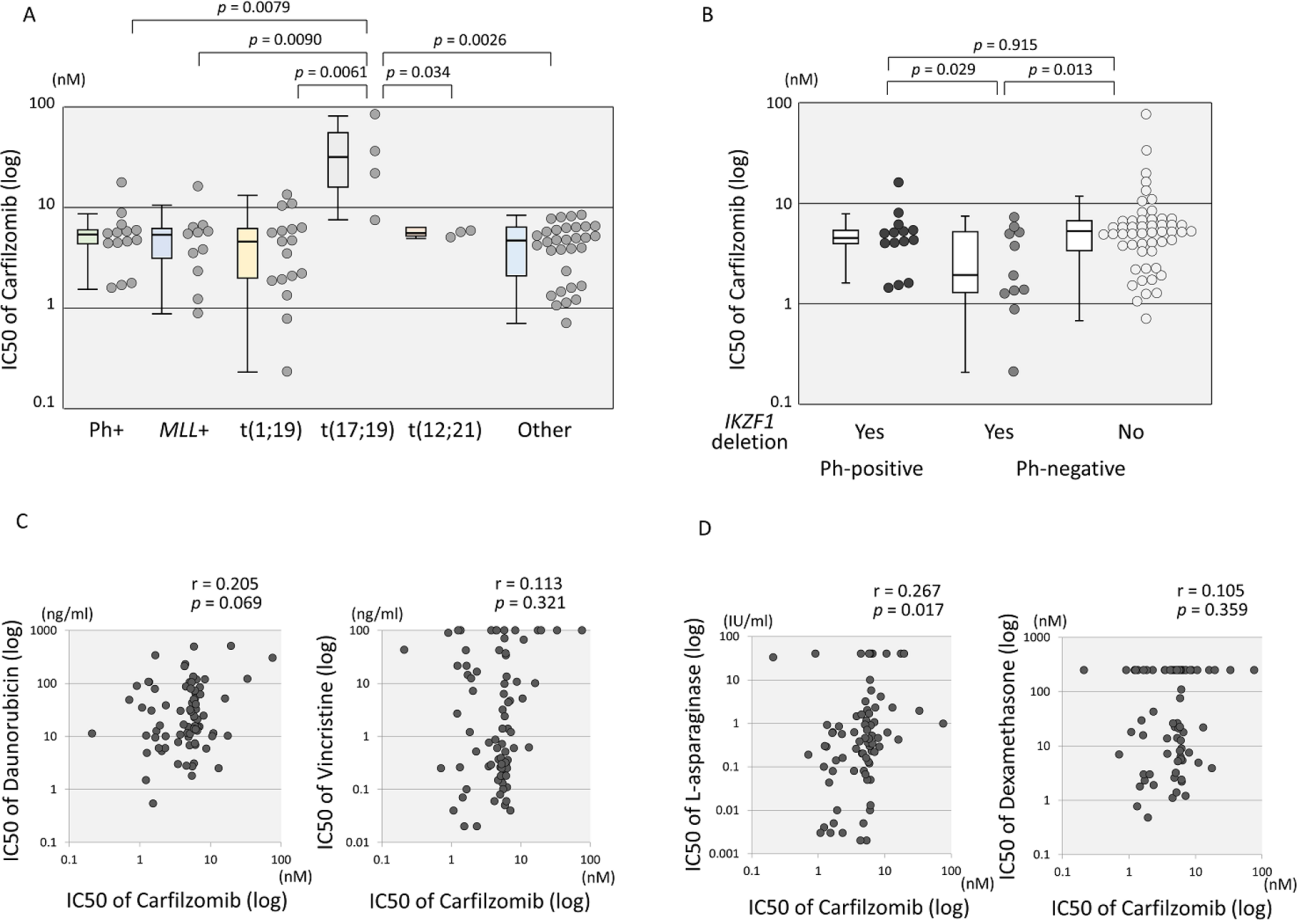
Anti-leukemic activity and cross-resistance between carfilzomib and four representative chemotherapeutic agents in BCP-ALL cell lines. (A) Association of carfilzomib sensitivity with different types of translocation in BCP-ALL cell lines. The vertical axis indicates the log IC50 value for carfilzomib. Boxplot of each type is indicated. The *p* value determined by a Mann-Whitney test is indicated when < 0.1. (B) Association of *IKZF1* deletion with carfilzomib sensitivity in Ph+ALL and Ph-negative BCP-ALL cell lines. The vertical axis indicates the log IC50 value for carfilzomib. The *p* value determined by a Mann-Whitney test is indicated. (C and D) Cross-resistance between carfilzomib and representative chemotherapeutic agents in 79 BCP-ALL cell lines. The vertical axes indicate the log IC50 values for daunorubicin (C), vincristine (C), L-asparaginase (D), and dexamethasone (D), whereas the horizontal axis indicates that for carfilzomib. Correlation coefficients and *p* values are shown at the top of each panel.

Among all the BCP-ALL cell lines (S2 Table and Fig 5A), the t(17;19)-ALL cell lines (median IC50: 26.5 nM; Mean ± SD: 33.9 ± 30.2 nM) were significantly more resistant to CFZ than the Ph+ALL (5.68 nM; 5.9 ± 4.0 nM; p < 0.01), *MLL*+ALL (5.36 nM; 5.2 ± 4.1 nM; p < 0.01), t(1;19)-ALL (4.66 nM; 5.1 ± 3.8 nM; p < 0.01), t(12;21)-ALL (5.81 nM; 5.6 ± 0.4 nM; p = 0.034), and B-other ALL cell lines (5.16 nM; 4.5 ± 2.2 nM; p < 0.01). Although deletion of *IKZF1* was not associated with higher CFZ sensitivity in 79 BCP-ALL cell lines including Ph+ALL cell lines, 11 Ph-negative BCP-ALL cell lines carrying *IKZF1* deletion (1.93 nM; 3.11 ± 2.42 nM) were significantly more sensitive to CFZ than 54 Ph-negative BCP-ALL cell lines without the deletion (5.53 nM; 7.35 ± 10.8 nM; p = 0.013) (Fig 5B). With regard to biallelic loss of *CDKN2A*, no significant association with CFZ sensitivity was observed in 79 BCP-ALL cell lines and in 65 Ph-negative BCP-ALL cell lines (data not shown). Finally, we examined the cross-resistance of CFZ to the four drugs that were used in the TACL study regimen using 79 BCP-ALL cell lines (Fig 5C and 5D). There was no significant correlation between the log IC50 values of CFZ and those of the four drugs except for a weak correlation (r = 0.267, p = 0.017) with those of L-Asp.

### Association of P-glycoprotein expression with resistance to CFZ

**Fig 6:**
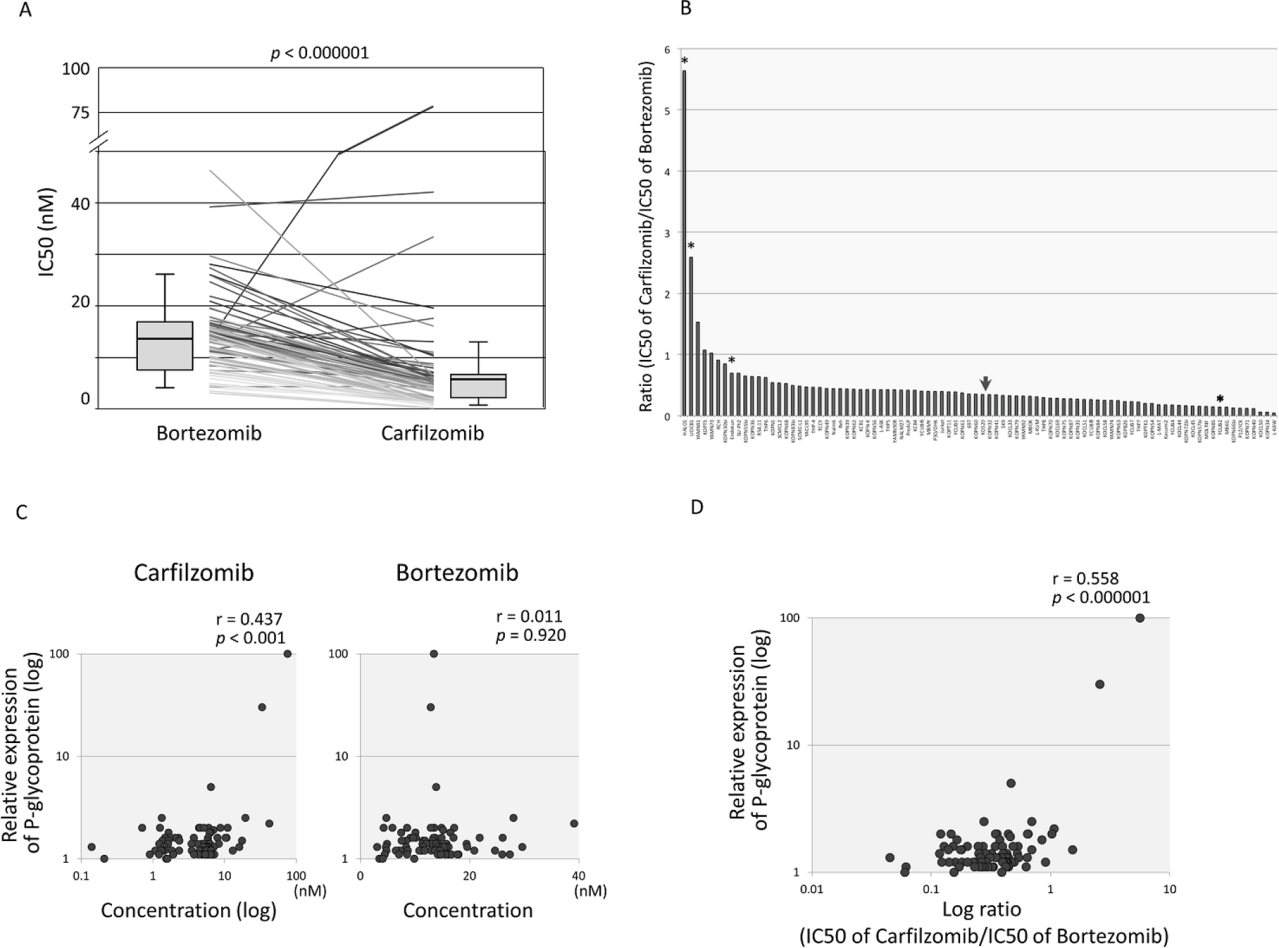
Involvement of P-glycoprotein in specific resistance to carfilzomib. (A) Comparison of sensitivities to carfilzomib and bortezomib in 79 BCP-ALL cell lines and 9 T-ALL cell lines. IC50 of bortezomib (left) and that of carfilzomib (right) in each cell line is connected by line. The vertical axis indicates concentrations of bortezomib and carfilzomib. Boxplots of 88 samples and the p-value in a paired t-test are indicated. (B) Distribution of ratio of bortezomib IC50 value to carfilzomib IC50 value in 79 BCP-ALL cell lines and 9 T-ALL cell lines. Arrowhead indicates median and asterisks indicate t(17;19)-ALL cell lines. (C) Correlation between cell surface expression level of P-glycoprotein and sensitivities to carfilzomib and bortezomib in 79 BCP-ALL cell lines and 9 T-ALL cell lines. The vertical axis indicates log value of relative P-glycoprotein expression, and the horizontal axes indicated log value of carfilzomib IC50 (left panel) or bortezomib IC50 (right panel). Correlation coefficients and *p* values are shown at the top of each panel. (D) Correlation between cell surface expression level of P-glycoprotein and sensitivities to carfilzomib and bortezomib in 79 BCP-ALL cell lines and 9 T-ALL cell lines. The vertical axis indicates the log value of relative P-glycoprotein expression, and the horizontal axis indicates the log ratio value of bortezomib IC50 to carfilzomib IC50. Correlation coefficient and *p* value are shown at the top of each panel.

In general, the anti-leukemic activity of CFZ was higher than that of BTZ, and the IC50 of CFZ was significantly lower than that of BTZ in 79 BCP-ALL cell lines and 9 T-ALL cell lines (p<0.000001 by paired t-test) (Fig 6A). The median IC50 of CFZ (5.3 nM; Mean ± SD: 6.57 ± 9.66 nM) was less than half of that of BTZ (13.5 nM; 13.8 ± 7.2 nM), and the median ratio of IC50 of BTZ to that of CFZ was 0.35 (Mean ± SD: 0.46 ± 0.65) (Fig 6B). Exceptionally, two cell lines were significantly more resistant to CFZ than BTZ; the ratio of IC50 of BTZ to IC50 of CFZ was 5.6 in HALO1 cells and 2.6 in UOCB1 cells. Both HALO1 and UOCB1 are t(17;19)-ALL cell lines, and a previous report [38] showed that both cell lines were positive for cell surface expression of P-glycoprotein (P-gp; ABCB1). The involvement of P-gp upregulation in resistance to CFZ was reported in MM cell lines [39, 40] and in lung and colon adenocarcinoma cell lines [41]. Thus, we investigated the cell surface expression level of P-gp in BCP-ALL and T-ALL cell lines using flow cytometry. P-gp expression was clearly detectable in HALO1 and UOCB1 cells, whereas it was marginal or undetectable in the other cell lines (S2 Table and S2 Fig). Cell surface expression level of P-gp tended to be correlated with IC50 of CFZ but not with that of BTZ (Fig 6C), and it was correlated with ratio of IC50 of BTZ to that of CFZ (Fig 6D).

### Effect of P-glycoprotein inhibitors on resistance to CFZ

**Fig 7:**
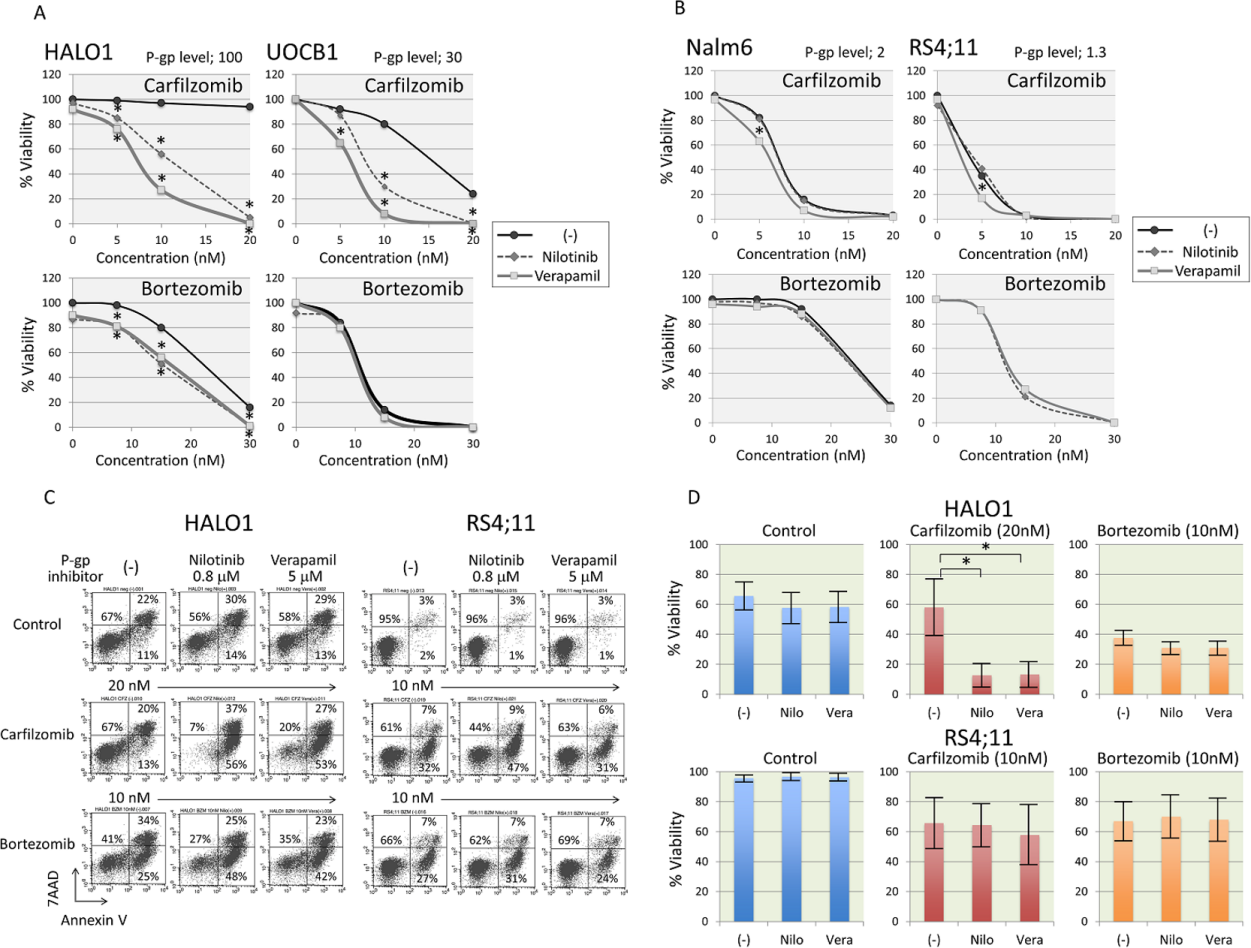
Effect of P-glycoprotein inhibitors on resistance to carfilzomib. (A and B) Effects of P-glycoprotein inhibitors on carfilzomib and bortezomib sensitivities in P-glycoprotein-positive (A) and P-glycoprotein-negative ALL cell lines (B). The vertical axes indicate % viability determined by alamarBlue cell viability assay and the horizontal axes indicate the concentration of carfilzomib (upper panel) or bortezomib (lower panel). Solid lines, dotted lines, and gray lines indicate dose response curves of carfilzomib or bortezomib alone, carfilzomib or bortezomib in combination with 0.8 μM of nilotinib, and carfilzomib or bortezomib in combination with 5 μM of verapamil, respectively. Mean values in triplicated analyses are indicated, and asterisk is indicated when p value in t-test is <0.05. Relative expression level of P-glycoprotein (P-gp) in each cell line is indicated at the top of panel. (C) Induction of apoptotic cell death by carfilzomib in combination with verapamil or nilotinib. HALO1 cells (left panels) and RS4;11 cells (right panels) were cultured with 20 nM (HALO1) or 10 nM (RS4;11) of carfilzomib (middle panels) or 10 nM of bortezomib (bottom panels) in the presence or absence of 0.8 μM of nilotinib or 5 μM of verapamil for 18 hours, and analyzed with Annexin V-binding (horizontal axis) and 7AAD-staining (vertical axis) using flow cytometry. The percentages of living cells (Annexin V-negative/7AAD-negative) and early (Annexin V-positive/ 7AAD-negative) and late (Annexin V-positive/ 7AAD-positive) apoptotic cells are indicated. (D) Effect of P-glycoprotein inhibitors on carfilzomib (middle panels) and bortezomib (right panels) sensitivities in P-glycoprotein-positive HALO1 (upper panel) and P-glycoprotein-negative RS4;11 (lower panel) cells. The vertical axis indicates cell viability determined by Annexin V-binding and 7AAD-staining in flow cytometric analyses. Mean ± SD of three independent experiments are indicated. Asterisks indicate significance (*p<0.05) in a paired t-test.

To further verify a possible involvement of P-gp expression in specific resistance to CFZ, we examined the sensitivities to CFZ and BTZ in the presence or absence of verapamil [42] or nilotinib [43], both of which are potent inhibitors of P-gp through different mechanisms. Indeed, treatment of P-gp-positive HALO1 cells with P-gp inhibitors significantly intensified the staining level of CAM, a P-gp sensitive dye, while treatment of P-gp-negative RS4;11 with P-gp inhibitors did not significantly affect the staining level of CAM (S3A and S3B Fig). Of note, P-gp-positive HALO1 and UOCB1 cells were dramatically sensitized to CFZ, but not to BTZ, in the presence of verapamil or nilotinib (Fig 7A), while sensitivities to CFZ and BTZ of P-gp-negative Nalm6 and RS4;11 cells were unchanged (Fig 7B). In P-gp-positive HALO1 cells, CFZ (20 nM) alone did not induce apoptosis, while CFZ in combination with verapamil or nilotinb, which alone showed minimal effect, clearly induced apoptosis (Fig 7C and 7D). P-gp inhibitors did not significantly affect the induction of apoptosis by BTZ (10nM) in HALO1 cells. In P-gp-negative RS4;11 cells, sensitivities to CFZ (10nM) and BTZ (10nM) were not significantly affected by P-gp inhibitors.

### Induction of resistance to CFZ by overexpression of P-glycoprotein

**Fig 8:**
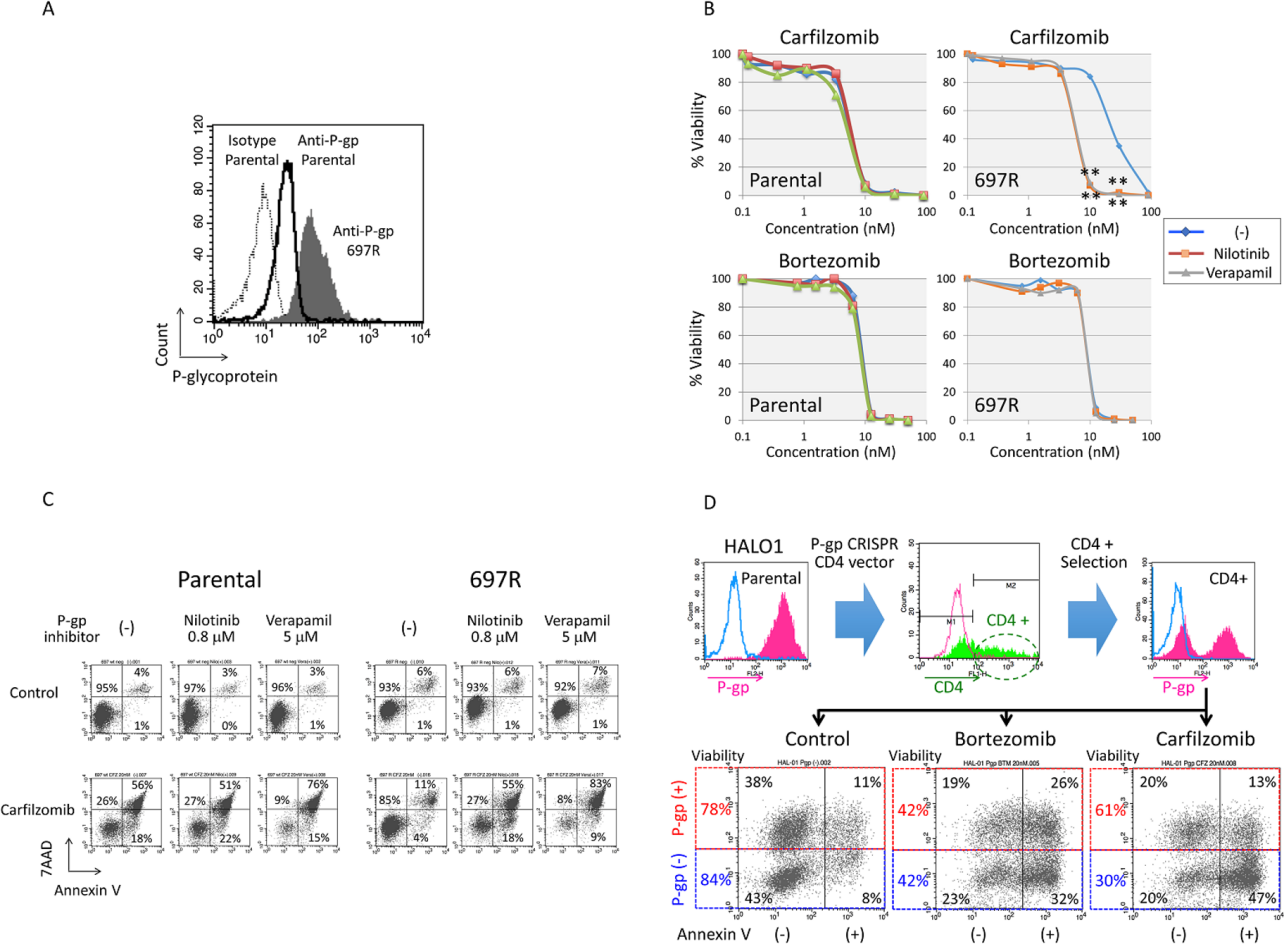
Effect of overexpression and knockout of P-glycoprotein on carfilzomib sensitivity. (A) Cell surface expression of P-glycoprotein in parental 697 cells and 697R cells. Dotted and solid lines indicate fluorescence intensities of isotype control and anti-P-glycoprotein (P-gp) antibodies in parental cells, respectively. Shade indicates fluorescence intensity of anti-P-glycoprotein antibody in 697R cells. (B) Effects of P-glycoprotein (P-gp) inhibitors on carfilzomib (upper panels) and bortezomib (lower panels) sensitivities in parental 697 cells (left panels) and 697R cells (right panels). The vertical axes indicate % viability determined by alamarBlue cell viability assay and the horizontal axes indicate the concentration of carfilzomib or bortezomib. Blue, red, and green lines indicate dose response curves of carfilzomib or bortezomib alone, carfilzomib or bortezomib in combination with 0.8 μM of nilotinib, and carfilzomib or bortezomib in combination with 5 μM of verapamil, respectively. Mean values in triplicated analyses are indicated, and asterisks are indicated when p value in t-test is <0.01. (C) Induction of apoptotic cell death by carfilzomib in combination with P-glycoprotein (P-gp) inhibitors. Parental 697 cells (left panels) and 697R cells (right panels) were cultured with 20 nM of carfilzomib in the presence or absence of 0.8 μM of nilotinib or 5 μM of verapamil for 18 hours, and analyzed with Annexin V-binding (horizontal axis) and 7AAD-staining (vertical axis) using flow cytometry. The percentages of living cells (Annexin V-negative/7AAD-negative) and early (Annexin V-positive/ 7AAD-negative) and late (Annexin V-positive/ 7AAD-positive) apoptotic cells are indicated. (D) Effect of P-glycoprotein knockout on bortezomib and carfilzomib sensitivities in HALO1 cells. P-glycoprotein-positive HALO1 cells were electroporated with an *ABCB1*-specific CRISPR/Cas 9 vector that contains cDNA of Cas9 fused to human CD4 cDNA via a 2A peptide sequence. 48 hours after electroporation, CD4-positive cells were harvested using anti-CD4 antibody. Upper middle panel indicates flow cytometric analysis of CD4 expression 3 days after electroporation. Upper left and upper right panels indicate flow cytometric analyses of P-glycoprotein (P-gp) expression in parental and CD4-positive population of HALO1 cells, respectively. Bottom panels indicate two color analysis of P-glycoprotein (P-gp) expression (vertical axis) and Annexin V-binding (horizontal axis) by flow cytometry in the CD4-positive populations of HALO1 cells treated with 20nM of bortezomib and 20nM of carfilzomib for 12 hours. Cell viabilities in P-glycoprotein (P-gp)-positive and negative populations are indicated at the left side of each panel.

To directly confirm an association of P-gp expression with specific resistance to CFZ, we first analyzed P-gp-positive subline of 697 cell line [31] that was established after long-term culture of 697 cells in the presence of stepwise increasing concentrations of silvestrol, a P-pg-sensitive inhibitor of protein synthesis. Silvestrol-resistant 697 subline (697R) was highly positive for cell surface expression of P-gp (Fig 8A) whereas parental 697 cells were weakly positive. Consistently, the level of CAM staining was significantly intensified in 697R cells by the treatment with P-gp inhibitors (nilotinib and verapamil) (S4A and S4B Fig), while that was almost unchanged in parental cells. Parental 697 cells and 697R cells were equally sensitive to BTZ in the alamarBlue cell viability assay (Fig 8B). In contrast, 697R cells were highly resistant to CFZ whereas parental 697 cells were sensitive. Of note, in the presence of P-gp inhibitors (nilotinib and verapamil), 697R cells were equally sensitive to CFZ as parental cells. In flow cytometric analysis of Annexin-V-binding and 7AAD-staining (Fig 8C), parental 697 cells were sensitive to CFZ regardless of the presence or absence of P-gp inhibitors. 697R cells were highly resistant to CFZ, and treatment with P-gp inhibitors sensitized 697R cells to CFZ.

### Restoration of sensitivity to CFZ by knockout of P-glycoprotein expression

Next, we tried to knock out P-gp expression in HALO1 using CRISPR/Cas9. An *ABCB1*-specific CRISPR/Cas9 vector that contains cDNA of Cas9 fused to human CD4 cDNA via a 2A peptide sequence was electroporated into HALO1 cells. 3 days after electroporation, CD4-positive cells were harvested using anti-CD4 antibody and subsequently expanded for further analysis. Flow cytometric analysis of P-gp expression (Fig 8D) demonstrated that P-gp expression was knocked out in nearly half of the CD4-positive population. Then, we treated the cells with either 20nM of BTZ or CFZ for 12 hours. Two color analysis of P-gp expression and Annexin V-binding (Fig 8D) revealed that P-gp-positive and P-gp-negative populations of HALO1 cells were equally sensitive to BTZ (cell viabilities: 42% vs. 42%). In contrast, P-gp-negative population was sensitive to CFZ (cell viability: 30%) whereas P-gp-positive population was resistant (61%). These observations directly indicated that P-gp expression is highly involved in the CFZ-specific resistance of HALO1 cells.

## Discussion

In the present study, Ph+ALL cell lines showed relatively higher sensitivity to BTZ in a large panel of BCP-ALL cell lines that acted as a model system for refractory ALL. This is consistent with a previous report in a mouse model of Ph+leukemia showing that BTZ stimulated proteasome-dependent degradation of FoxO3a, which is involved in leukemogenesis of Ph+leukemia since it is one of the downstream effectors of BCR-ABL [44]. Our observations suggested that *IKZF1* deletion and biallelic loss of *CDKN2A* may be additional factors that contribute to higher BTZ sensitivity in Ph+ALL. As observed in the patient’s samples [36, 37], Ph+ALL cell lines frequently harbored *IKZF1* deletion and biallelic loss of *CDKN2A* in the present study. Of note, even in Ph-negative BCP-ALL cell lines, *IKZF1* deletion and biallelic loss of *CDKN2A* were independently associated with higher BTZ sensitivity, suggesting that frequent association of *IKZF1* deletion and biallelic loss of *CDKN2A* may somehow contribute to the higher BTZ sensitivity of Ph+ALL cell lines. Although we could not clarify the underlying mechanisms that govern the involvement of *IKZF1* deletion and biallelic loss of *CDKN2A* in higher BTZ sensitivity, it should be noted that the underlying mechanisms that contribute to poor therapeutic outcome in ALL with *IKZF1* deletion remain to be elucidated [45]. The mechanisms that regulate the association of *IKZF1* deletion with higher BTZ sensitivity may overlap at least in part with those that are involved in poor response to conventional chemotherapy. *IKZF1* deletion [46] and biallelic loss of *CDKN2A* [47] are poor prognostic factors in ALL and are more frequently observed in refractory ALL patients than in newly diagnosed ALL cases [48]. Thus, a significant proportion of refractory ALL patients in the TACL study [8, 9] may have harbored *IKZF1* deletion and/or biallelic loss of *CDKN2A*, although the gene copy number alteration profile was not described. Our findings suggest that association of *IKZF1* deletion and biallelic loss of *CDKN2A* with higher BTZ sensitivity may have been a factor that contributed to favorable outcomes in refractory ALL patients treated with BTZ combination therapy in the TACL study at least in part.

In the TACL study, significance of BTZ combination chemotherapy in T-ALL patients was inconclusive, since only two patients were enrolled [9]. The present study revealed that T-ALL cell lines were relatively more sensitive to BTZ and CFZ than BCP-ALL cell lines. Consistently, Szczepanek *et al* [49] reported that the *in vitro* sensitivity to BTZ was significantly higher in T-ALL than BCP-ALL samples from pediatric ALL patients. Previous reports revealed frequent biallelic loss of *CDKN2A* in samples from T-ALL patients [50, 51], which suggested that there is an association between higher *in vitro* BTZ sensitivity of T-ALL and frequent biallelic loss of *CDKN2A*. In contrast, Szczepanek *et al* [49] reported that T-ALL samples were significantly more resistant to most of the chemotherapeutic agents *in vitro* than BCP-ALL samples. Further studies are required to verify the efficacy of BTZ combination chemotherapy in T-ALL patients.

In our series of BCP-ALL cell lines, sensitivity to L-Asp showed moderate, weak, and marginal correlation with that to VCR, Dex, and DNR, respectively, and sensitivity to DNR showed weak correlation with that to VCR, which suggested that there is cross-resistance between multiple agents for ALL treatment. In contrast, BTZ did not show any significant cross-resistance to the four drugs except for a weak cross-resistance to L-Asp. The observed minimal cross-resistance between BTZ and conventional chemotherapeutic agents in cell lines is consistent with the efficacy of BTZ combination chemotherapy in refractory BCP-ALL patients. A significant proportion of refractory ALL patients are intolerable to Asp due to allergic response, and pancreatitis [52]. Thus, in refractory ALL patients, elimination of Asp could be beneficial for safer efficacy of BTZ combination chemotherapy. Recently, Yeo *et al* [53] reported the efficacy and safety of BTZ combination therapy with Dex, mitoxantrone, and vinorelbine for relapsed childhood ALL patients who were intolerant to Asp. Of note, without Asp, seven of 10 patients achieved CR after one cycle of therapy, and four patients achieved negative minimal residual disease. Taken together, the BTZ combination chemotherapy without Asp could be an effective and safer therapeutic option for refractory ALL patients.

To further improve the outcome of BTZ combination chemotherapy in refractory ALL patients, substitution of BTZ by CFZ is attractive considering the durable and irreversible activity of CFZ [10]. In the present study, the anti-leukemic activity of CFZ was higher than that of BTZ in most of the BCP-ALL and T-ALL cell lines, and *IKZF1* deletion was also associated with higher CFZ sensitivity in Ph-negative BCP-ALL cell lines. In clinical settings, Dex combination therapy with CFZ revealed significantly better outcome than that with BTZ in a randomized phase 3 study for relapsed MM patients [17, 18]. Thus, although additional studies are required to confirm its safety, CFZ combination chemotherapy seems to be a promising new therapeutic option for refractory ALL patients. However, in the present study, two t(17;19)-ALL cell lines showed specific resistance to CFZ. Previous reports indicated that acquired CFZ-resistant sublines showed marked upregulation of P-glycoprotein in comparison with their parental cell lines [40, 41]. Indeed, we confirmed that two t(17;19)-ALL cell lines that showed specific resistance to CFZ clearly overexpressed P-glycoprotein as previously reported [38]. Consistently, restoration of cellular sensitivity to CFZ was induced by co-incubation with verapamil [42] or nilotinib [43], which are P-glycoprotein inhibitors. Furthermore, we confirmed that overexpression of P-glycoprotein was associated with specific resistance to CFZ in 697R cells [31] and that inhibitors for P-glycoprotein sensitized 697R cells to CFZ. We also revealed that knockout of P-glycoprotein in P-glycoprotein-positive HALO1 cells by CRISPR/Cas9 system sensitized HALO1 cells to CFZ. These observations demonstrated that overexpression of P-glycoprotein plays a major role in specific resistance to CFZ. Previous reports revealed that overexpression of P-glycoprotein in ALL is associated with poor prognosis and is more common in relapsed patients than in newly diagnosed patients [54–57]. Thus, application of CFZ combination chemotherapy for refractory ALL patients must be limited to the patients whose ALL cells are negative for cell surface expression of P-glycoprotein.

In conclusion, although there was a limitation in interpretation of the results due to cell line study, we demonstrated that BTZ combination regimen without Asp may be a safe and effective therapy for refractory ALL, and substitution of BTZ with CFZ may further improve the clinical outcome except in cases with P-glycoprotein overexpression.

## Supporting information

S1 Fig Distributions of *IKZF1* deletion and homozygous *CDKN2A* deletion in Ph+ALL cell lines and in Ph-negative ALL cell lines. (TIF)

S2 Fig Cell surface expression of P-glycoprotein. Shade indicates fluorescence intensity of anti-P-glycoprotein antibody, and line indicates that of control antibody. Relative fluorescence intensity of each cell line is indicated in the middle of each panel, and ratio of bortezomib IC50 value to carfilzomib IC50 value of each cell line is indicated at the top of each panel.
(TIF)

S3 Fig Effect of P-glycoprotein inhibitors on CAM staining. (A) Flow cytometric analysis of CAM staining in P-glycoprotein-positive HALO1 cells (left panels) and P-glycoprotein-negative RS4;11 cells (right panels) cultured in the presence or absence of P-glycoprotein (P-gp) inhibitors (0.8 μM of nilotinib or 5 μM of verapamil). Geometric mean (GeoMean) of CAM staining is indicated in each panel. (B) Effect of P-glycoprotein (P-gp) inhibitors on CAM staining of P-glycoprotein-positive HALO1 cells (left panel) and P-glycoprotein-negative RS4;11 cells (right panel). The vertical axis indicates GeoMean of CAM staining. Mean ± SD of triplicated experiments are indicated. Asterisks indicate significance (**p<0.01) in a paired t-test.
(TIF)

S4 Fig Effect of overexpression of P-glycoprotein on CAM staining. (A) Flow cytometric analysis of CAM staining in parental 697 cells (left panels) and 697R cells (right panels) cultured in the presence or absence of P-glycoprotein (P-gp) inhibitors (0.8 μM of nilotinib or 5 μM of verapamil). Geometric mean (GeoMean) of CAM staining is indicated in each panel. (B) Effect of P-glycoprotein (P-gp) inhibitors on CAM staining of parental 697 cells (left panels) and 697R cells (right panels). The vertical axis indicates GeoMean of CAM staining. Mean ± SD of triplicated experiments are indicated. Asterisks indicate significance (**p<0.01, *0.01<p<0.05) in a paired t-test.
(TIF)

S1 Table List of cell lines. (TIF)

S2 Table Summary of data. (TIF)

S3 Table Association of *IKZF1*, and *CDKN2A/CDKN2B* deletion. (TIF)

S4 Table Cross-resistance among BTZ, DNR, VCR, L-Asp, and Dex in 79 BCP-ALL cell lines. (TIF)
