# Supplementary figures and images for: Anti-leukemic activity of bortezomib and carfilzomib on B-cell precursor ALL cell lines
Source: PLoS One. 2017 Dec 13;12(12):e0188680. doi: 10.1371/journal.pone.0188680 (PMC5728482; doi:10.1371/journal.pone.0188680)

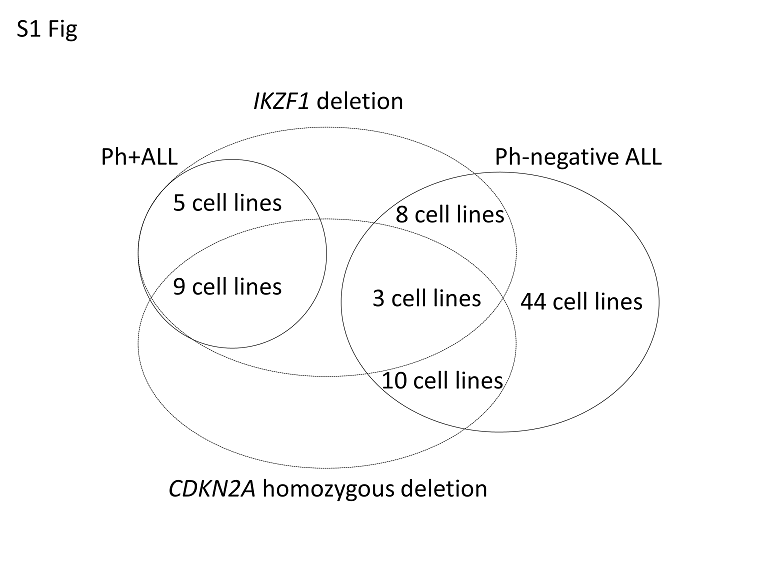

Supplement: S1 Fig — (TIF) [file pone.0188680.s001.tif]

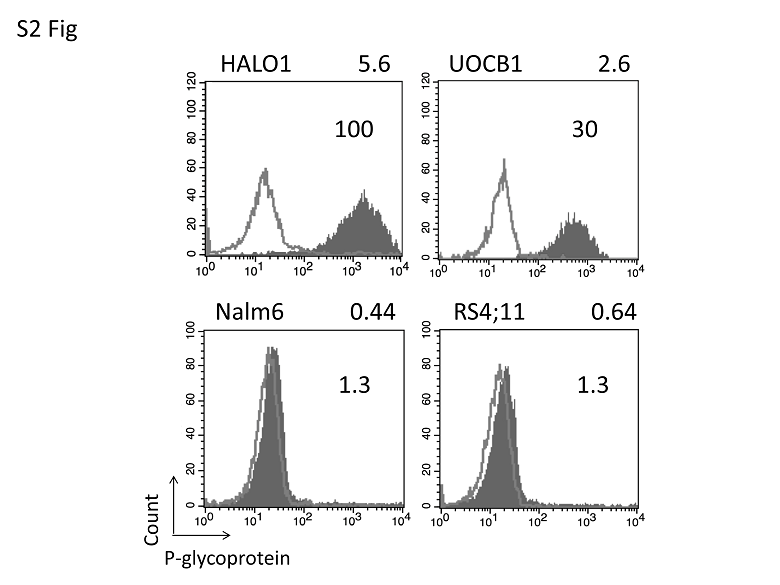

Supplement: S2 Fig — Shade indicates fluorescence intensity of anti-P-glycoprotein antibody, and line indicates that of control antibody. Relative fluorescence intensity of each cell line is indicated in the middle of each panel, and ratio of bortezomib IC50 value to carfilzomib IC50 value of each cell line is indicated at the top of each panel. (TIF) [file pone.0188680.s002.tif]

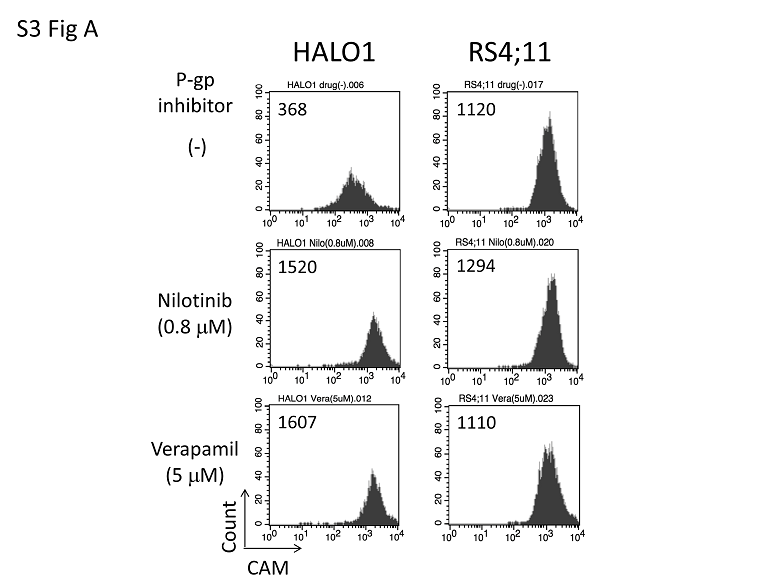

Supplement: S3 Fig — (A) Flow cytometric analysis of CAM staining in P-glycoprotein-positive HALO1 cells (left panels) and P-glycoprotein-negative RS4;11 cells (right panels) cultured in the presence or absence of P-glycoprotein (P-gp) inhibitors (0.8 μM of nilotinib or 5 μM of verapamil). Geometric mean (GeoMean) of CAM staining is indicated in each panel. (B) Effect of P-glycoprotein (P-gp) inhibitors on CAM staining of P-glycoprotein-positive HALO1 cells (left panel) and P-glycoprotein-negative RS4;11 cells (right panel). The vertical axis indicates GeoMean of CAM staining. Mean ± SD of triplicated experiments are indicated. Asterisks indicate significance (**p<0.01) in a paired t-test. (TIF) [file pone.0188680.s003.tif]

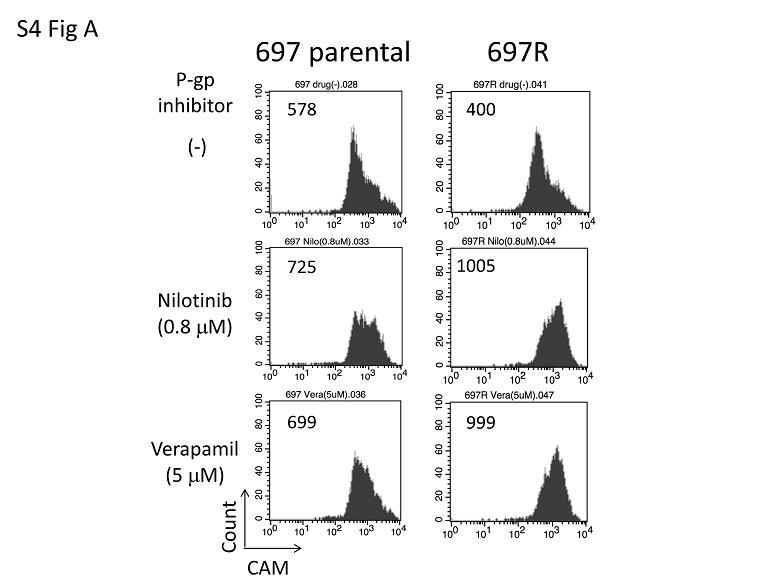

Supplement: S4 Fig — (A) Flow cytometric analysis of CAM staining in parental 697 cells (left panels) and 697R cells (right panels) cultured in the presence or absence of P-glycoprotein (P-gp) inhibitors (0.8 μM of nilotinib or 5 μM of verapamil). Geometric mean (GeoMean) of CAM staining is indicated in each panel. (B) Effect of P-glycoprotein (P-gp) inhibitors on CAM staining of parental 697 cells (left panels) and 697R cells (right panels). The vertical axis indicates GeoMean of CAM staining. Mean ± SD of triplicated experiments are indicated. Asterisks indicate significance (**p<0.01, *0.01<p<0.05) in a paired t-test. (TIF) [file pone.0188680.s004.tif]

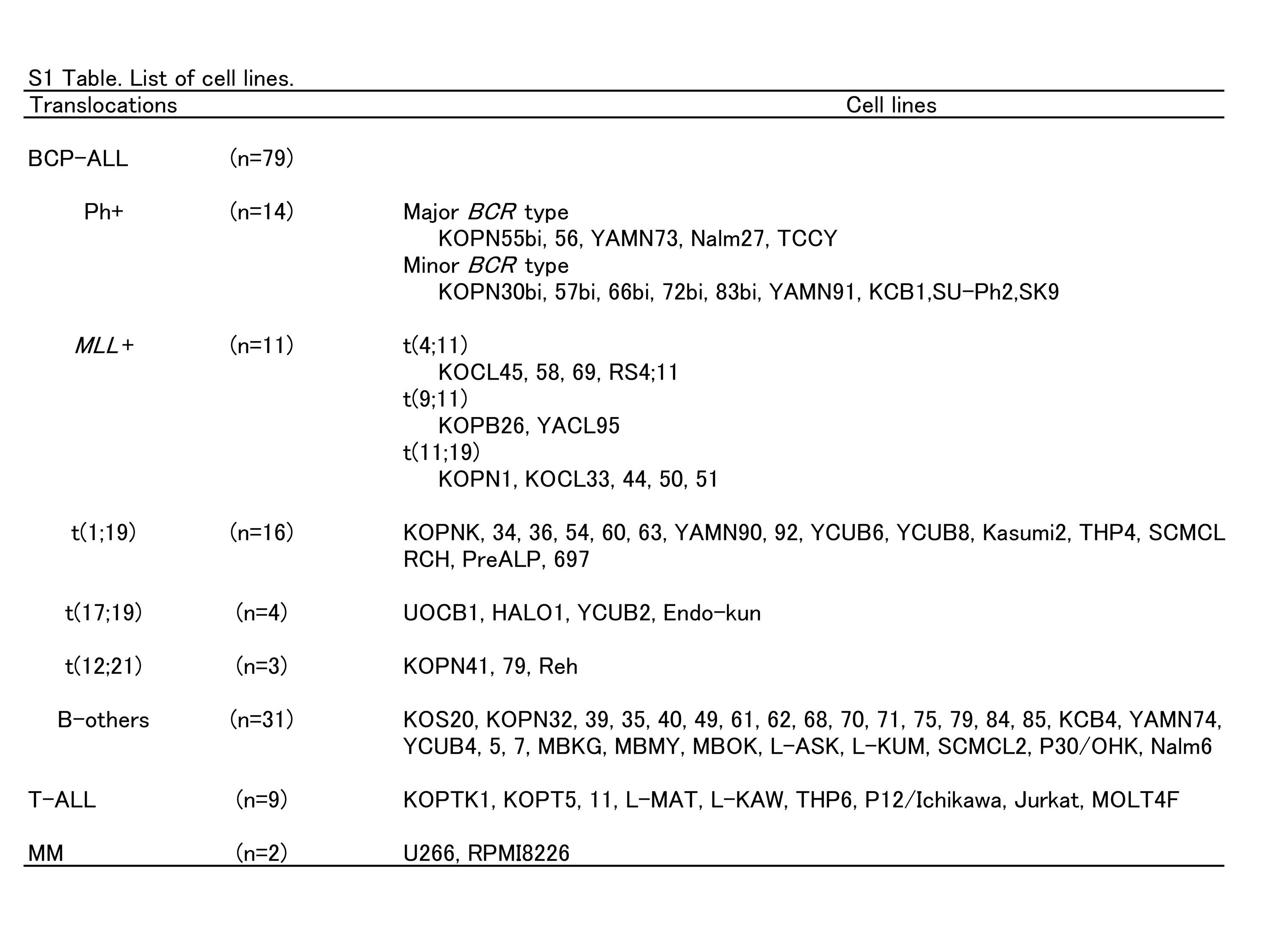

Supplement: S1 Table — (TIF) [file pone.0188680.s005.tif]

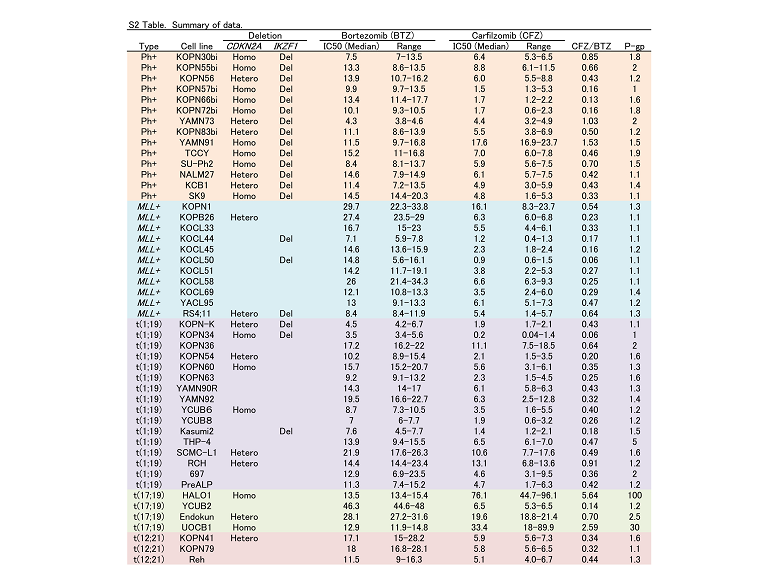

Supplement: S2 Table — (TIF) [file pone.0188680.s006.tif]

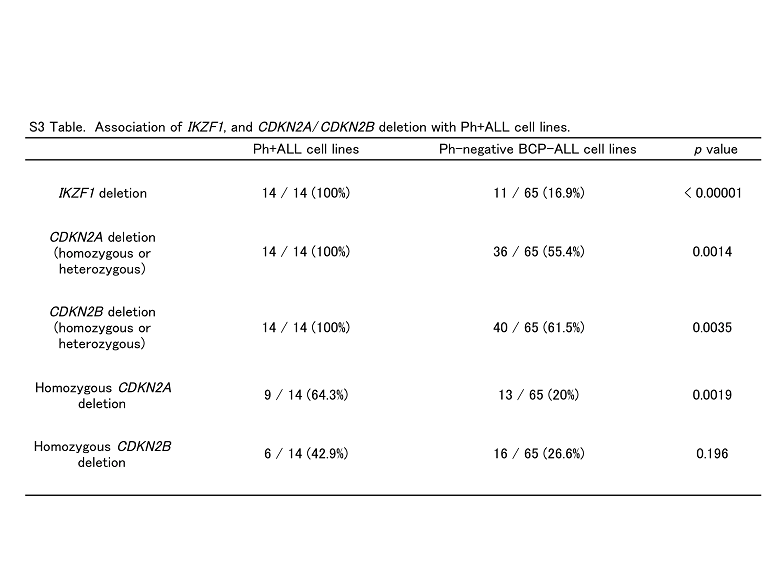

Supplement: S3 Table — (TIF) [file pone.0188680.s007.tif]

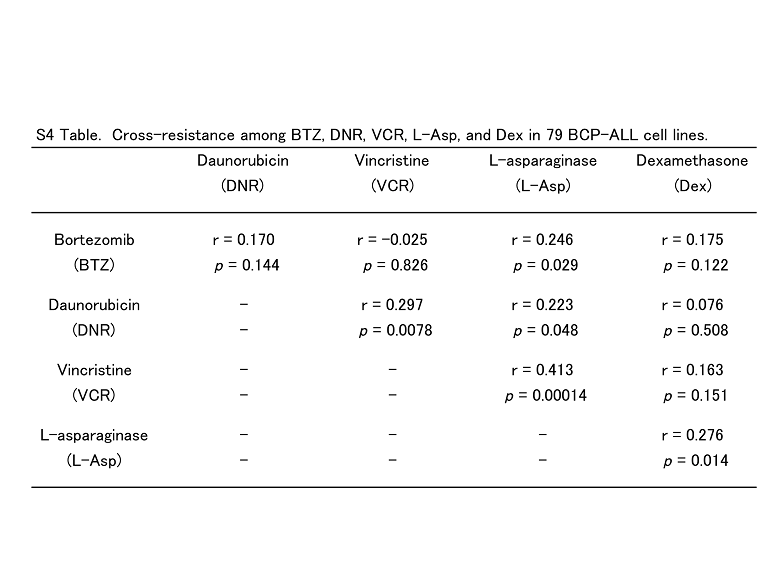

Supplement: S4 Table — (TIF) [file pone.0188680.s008.tif]
